# Down in the pond: Isolation and characterization of a new *Serratia marcescens* strain (LVF3) from the surface water near frog’s lettuce (*Groenlandia densa*)

**DOI:** 10.1371/journal.pone.0259673

**Published:** 2021-11-08

**Authors:** Ines Friedrich, Bernhard Bodenberger, Hannes Neubauer, Robert Hertel, Rolf Daniel

**Affiliations:** 1 Genomic and Applied Microbiology & Göttingen Genomics Laboratory, Institute of Microbiology and Genetics, Georg-August-University of Göttingen, Göttingen, Germany; 2 FG Synthetic Microbiology, Institute of Biotechnology, BTU Cottbus-Senftenberg, Senftenberg, Germany; Academia Sinica, TAIWAN

## Abstract

*Serratia marcescens* is a species that belongs to the family of *Yersiniaceae*. This family comprises taxa representing opportunistic human- and phytopathogens but also plant growth-promoting rhizobacteria (PGPR). This study describes a novel Gram-negative strain (LVF3^R^) of the species *Serratia marcescens*. The strain was characterized genomically, morphologically, and physiologically. In addition, the potential of the isolate to act as a host strain to assess the diversity of *Serratia* associated phages in environmental samples was explored. Average nucleotide identity analysis revealed that LVF3^R^ belongs to the species *Serratia marcescens*. *In silico* analysis and ProphageSeq data resulted in the identification of one prophage, which is capable of viral particle formation. Electron microscopy showed cells of a rod-shaped, flagellated morphotype. The cells revealed a length and width of 1–1.6 μm and 0.8 μm, respectively. LVF3^R^ showed optimal growth at 30 C and in the presence of up to 2% (w/v) NaCl. It exhibited resistances to ampicillin, erythromycin, oxacillin, oxytetracycline, rifampicin, tetracycline, and vancomycin. Genome data indicate that strain *S*. *marcescens* LVF3^R^ is a potential PGPR strain. It harbors genes coding for indole acetic acid **(**IAA) biosynthesis, siderophore production, plant polymer degradation enzymes, acetoin synthesis, flagellar proteins, type IV secretion system, chemotaxis, phosphorous solubilization, and biofilm formation.

## Introduction

The genus *Serratia* belongs to the order Enterobacterales, which is part of the Gammaproteobacteria, a large and diverse group of facultatively anaerobic, non-spore-forming, Gram-negative, rod-shaped bacteria. Related families are *Budviciaceae*, *Enterobacteriaceae*, *Erwiniaceae*, *Hafniaceae*, *Morganellaceae*, *Pectobacteriaceae* and *Yersiniaceae* [1]. The genus *Serratia* is part of the family *Yersiniaceae*, consisting of the eight genera *Chania*, *Chimaeribacter*, *Ewingella*, *Rahnella*, *Rouxiella*, *Samsonia*, *Serratia* and *Yersinia* [1]. *Yersiniacea* members are described as motile, catalase-positive and unable to produce hydrogen disulfide [1]. To date, the genus *Serratia* consists of 24 species (LPSN [2] accessed on 28 January 2021), which can be isolated from diverse environments such as soil, plants, animals, insects, and water [3,4].

The genus *Serratia* is named after the Italian physicist Serafino Serrati and was first discovered in 1819 by Bartolomeo Bizio in Padua, Italy. However, the history of *Serratia* reaches back to the Middle Ages when it played a role in eucharist miracles. Some *Serratia* strains produce a red and non-diffusible pigment designated prodigiosin. As they are able to grow on bread, these *Serratia* may have been used to mimic blood on church bread at the time [5]. *Serratia* cells are Gram-negative and rod-shaped with rounded ends, and do not form endospores [3], except the potential spore-forming *Serratia marcescens* subsp. *Sakuensis* [6]. However, the International Committee on Systematics of Prokaryotes has not yet been able to confirm this [4].

*Serratia* is frequently associated with animals and plants. It can be isolated from healthy individuals [3] and is associated with conjunctivitis in horses, septicemia in foals, pigs and goats, and mastitis in cows [7,8]. Some strains are opportunistic pathogens causing pneumonia, septicemia, or cutaneous lesions [9,10]. *Serratia marcescens* account for 1–2% of nosocomial infections in humans, mostly occurring in the respiratory or urinary tract, surgical wounds, and soft tissues [11–13]. On plants, *Serratia marcescens* strains can cause the cucurbit yellow vine disease (CYVD) in watermelons, pumpkins, and yellow squash, as well as soft-rot disease in the bell pepper [14–16]. Nevertheless, reports of plant-promoting *S*. *marcescens* strains also exist [17,18].

*Serratia* strains can produce industrially relevant extracellular enzymes such as highly active DNA/RNA endonucleases, lipases, proteinases and chitinases [3,19]. The pigment prodigiosin has antibacterial and antitumor properties and is produced by *S*. *marcescens*, *S*. *plymuthica* and *S*. *rubidaea* [3,20,21]. As *Serratia* species exhibit multiple antibiotic resistances, there is now a revival of interest in phages as therapeutic agents [22].

Phages or bacteriophages are viruses of bacteria. Lytic phages reproduce directly after infection, while temperate phages can integrate into the bacterial genome. There they inactivate, and replicate together with their host, resulting in a prophage and a lysogenic bacterium. A prophage can impart new properties to its host through the addition of its genetic material, thereby protecting it from infection with related and unrelated viruses [23].

Active *Serratia* bacteriophages can frequently be found in rivers and sewage [24–26]. *Serratia* phages are often able to infect related genera [27–29]. Lysogeny can frequently be observed within the genus *Serratia* [3]. To date, the complete genomic sequences of 14 *Serratia*-associated phages are available (accessed on 28 January 2021) in the NCBI Viral RefSeq database [30]. In order to isolate novel phages from the environment, safe and well-characterized host strains are required. Ideally, these should be non-pathogenic and have no or only few prophages to avoid prophage-induced resistance, which would lead to a strain which cannot be infected by phages.

In a previous study, we succeeded to isolate an environmental *Serratia marcescens* strain which originated from an oligotrophic pond in Göttingen, Germany (51° 33’ 59" N 9° 56’ 22" E 230 m, collected on 18 September 2018). The *Serratia* strain was isolated as potential model strain to study the local viral diversity associated with it. While 16S rRNA gene analysis confirmed its species assignment, no further characterization has been done previously [31].

In this study, an environmental *Serratia marcescens* isolate is characterized morphologically, physiologically and genomically. In addition, its potential as a host strain to access the environmental diversity of *Serratia* associated phages is explored.

## Material and methods

### Isolation of *Serratia marcescens* LVF3 strain, DNA extraction, and 16S rRNA gene sequencing

*Serratia marcescens* LVF3^R^ was isolated from the surface water near frog’s lettuce (*Groenlandia densa*) from an oligotrophic pond located in the northern part of Weende, Göttingen, Germany [31]. In this study, no specific permissions were required for the location, which is a public pond in Göttingen outside of any protected area. As culture medium, 25 mL TSB-10 (1.7% peptone from casein, 0.3% peptone from soybean, 0.25% K_2_HPO_4_, 1% NaCl, 0.25% glucose monohydrate) were used. DNA was extracted as described by Friedrich et al., 2021 [31].

### Genome and prophage sequencing, assembly, and annotation

The genome and prophages were sequenced, assembled and annotated as described in Friedrich et al. 2021. In brief, Illumina paired-end sequencing libraries were prepared using the Nextera XT DNA Sample Preparation kit and sequenced using the MiSeq System and Reagent Kit version 3 (2 x 300 bp) according to the manufacturer’s recommendations (Illumina, San Diego, CA, USA) [31]. For Nanopore sequencing, the Ligation Sequencing Kit (SQK-LSK109) and the Native Barcode Expansion Kit EXP-NBD114 (Barcode 14; Oxford Nanopore Technologies, Oxford, UK) were used [31].

Potential CRISPR regions were identified with CRISPRFinder [32]. Assembled genomes were quality-checked with CheckM v1.1.2 [33]. Genome annotation was performed by the NCBI (National Centre for Biotechnological Information) using the Prokaryotic Genome Annotation Pipeline v4.13 (PGAP) [34].

The whole-genome sequence of *Serratia marcescens* LVF3^R^ has been deposited at GenBank under the accession numbers CP063229 (chromosome) and CP063230 (plasmid). The BioProject with the accession number PRJNA669584 contains the BioSample SAMN16456043. The raw reads have been deposited in the NCBI SRA database under the accession numbers SRR12951277 (Oxford Nanopore) and SRR12951278 (Illumina MiSeq) and BioProject PRJNA669584. The strain has been deposited at the DSMZ (Deutsche Sammlung von Mikroorganismen und Zellkulturen, Braunschweig, Germany) under collection number DSM 112280.

### Phylogenetic classification of *Serratia marcescens* LVF3^R^

To provide an initial taxonomic classification of the *Serratia marcescens* isolate, the Genome Taxonomy Database Toolkit (GTDB-Tk) v1.0.2 [35] was used as well a whole-genome-based phylogeny with Type (Strain) Genome Server (TYGS [36], accessed on 31 January 2021). In-depth phylogenetic analysis was done with the ANIm method included in pyani v0.2.10 [37]. A species boundary of 95% ANI was used [35]. The isolate was compared to all available type strain and reference genomes based on the lists of the DSMZ and the NCBI (accessed on 28 April 2021): *Enterobacter asburiae* ATCC 35953^T^ (PRJNA285282), *Kluyvera cryocrescens* NBRC 102467^T^ (PRJDB285), *Raoultella planticola* ATCC 33531^T^ (PRJNA65511), *Raoultella planticola* DSM 2688^R^ (PRJNA500331), *Serratia ficaria* NBRC 102596^T^ (PRJDB1514), *S*. *inhibens* S40^T^ (PRJNA491277), *S*. *liquefaciens* ATCC 27592^T^ (PRJNA208332), *S*. *marcescens* ATCC 13880^T^ (PRJNA59561), *S*. *marcescens* subsp. *sakuensis* KCTC 42172^T^ (PRJNA484649), *S*. *nematodiphila* DSM 21420^T^ (PRJNA257492), *S*. *plymuthica* NBRC 102599^T^ (PRJDB268), *S*. *proteamaculans* CCUG 14510^T^ (PRJNA563568), *S*. *quinivorans* NCTC 11544^T^ (PRJEB6403), *S*. *rubidae* NBRC 103169^T^ (PRJDB269), *Serratia* sp. S119^R^ (PRJNA342012) and *Skermanella stibiiresistens* SB22^T^ (PRJNA214805).

### Comparative genomics

Metabolic capabilities of LVF3^R^ were investigated using BlastKOALA v2.2 [38] (S2 Fig). Putative secondary metabolite biosynthetic gene clusters were identified with antiSMASH v6.0.0b [39,40]. Putative phage regions were identified with PHASTER [41]. Antibiotic resistance annotation was investigated through Resfams v1.2.2 [42].

### Cell morphology and Gram staining procedure

Colony morphology was studied by microscopy (Primo Star, Zeiss, Carl Zeiss Microscopy, Jena, Germany) of single colonies (4X magnification) after growth on TSA-10 solid medium (Fluka, Munich, Germany) for 24 h. A Gram staining analysis was performed using Hucker’s crystal violet, an iodine and safranin solution and 1-propanol [43]. Microscopy images and staining were processed and evaluated with the software ZEISS Labscope (Carl Zeiss).

### Transmission electron microscopy

Cell morphology of LVF3^R^ was assessed by transmission electron microscopy (TEM). Data were imaged onto the screen using the digital Micrograph software (Gatan GmbH, Munich, Germany). The isolate was grown in liquid TSB-10 medium overnight at 30°C. Afterwards, a negative staining technique was performed. For this purpose, 5 μL cell suspension were mixed with the same amount of diluted 0.5% phosphotungstic acid (3% stock, pH 7) and were transferred to a vaporized carbon mica for 1 min. The mica was washed briefly with demineralized water and transferred to a thin copper-coated grid (PLANO GmbH, Marburg, Germany). The coated grids were dried at room temperature and examined through a Jeol 1011 TEM (Georgia Electron Microscopy, Freising, Germany).

### Determination of salt tolerance and temperature optimum

For the determination of the salt tolerance, LVF3^R^ was inoculated in 4 mL TSB medium amended with 0, 5 and 10 to 100 g/L NaCl in increments of 10 g. The optical density of the cell suspensions was measured using the Ultraspec 3300 pro photometer (Amersham Pharmacia Biotec Europe GmbH, Munich, Germany) at a wavelength of 600 nm (OD_600_). OD_600_ of the cell suspensions were set to 0.3 at the beginning of the experiment [44], followed by an incubation period of 3 h at 30°C and 180 rpm in a Infors HT shaker (Orbitron, Einsbach, Germany). After 3 h incubation, the OD_600_ was measured and the initial OD subtracted to assess growth [44]. All measurements were performed in biological replicates.

To quantify the temperature optimum, the isolate was grown in 4 mL TSB-10 medium at 10°C, 20°C, 30°C, 37°C, 40°C and 50°C at 180 rpm. The starting OD_600_ of the cell cultures was set to 0.1. The optical cell density of LVF3^R^ was measured after 3 h. The collected data was illustrated with R studio version 4.0.0 [45] using ggplot2 package [46].

### Determination of growth kinetics

The growth kinetics in liquid cultures were measured with the cell growth quantifier (CGQuant 8.1) (Aquila Biolabs GmbH, Baesweiler, Germany) at 30°C for 47 h. 25 mL of LVF3^R^ with a final OD_600_ of 0.1 in TSB-10 medium were filled into 250 mL shake flasks. All flasks were mounted onto the CGQuant sensor plate and were shaken for 47 h. The CGQuant enables a dynamic approach of backscattered light measurement, monitoring the growth of the liquid cultures in real-time [47]. All measurements were performed as biological replicates. All collected data were illustrated with R studio version 4.0.0 [45] using ggplot2 package [48].

### Metabolic activity and antibiotic resistances

Metabolic activities were identified using API ZYM and API 20 E tests (BioMérieux, Nuertingen, Germany). Both tests were performed according to the instructions of the manufacturer. Catalase activity was determined using 3% H_2_O_2_ [49]. For determination of antibiotic resistances, a soft-agar (0.4% (w/v) agarose in TSA-10 medium) overlay technique was used with discs, and strips (Oxoid, Thermo Fisher Scientific) containing ampicillin (25 μg), chloramphenicol (30 μg), doxycycline (30 μg), erythromycin (10 μg), kanamycin (30 μg), oxytetracycline (30 μg), rifampicin (2 μg), streptomycin (10 μg), vancomycin (30 μg), meropenem (0.002–32 μg), and oxacillin (0.015–256 μg). Soft agar (2.5 mL) was used to inoculate the isolates with a final OD_600_ of 0.1. Afterwards, discs or strips were placed on the soft agar. All plates were incubated overnight at 30°C.

### Plaque assay with sewage water

For phage enrichment, the same procedure was conducted as described by Willms & Hertel, 2016 [50] and Willms et al., 2017 [51]. After incubation, different plaque morphologies such as clear or turbid, the size of plaques, and the presence or absence of a halo were differentiated. Generally, the performance of a plaque assay requires the ability of the host to grow in bacterial lawns [52].

## Results and discussion

### Morphological characterization

**Fig 1:**
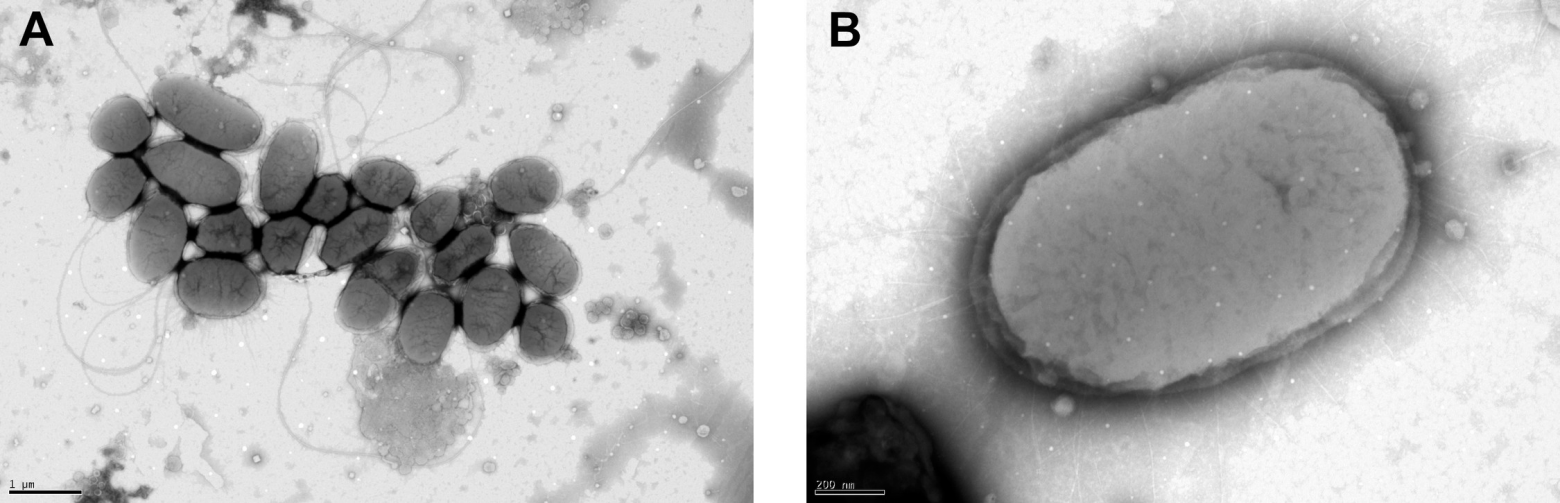
Transmission electron microscopy images of LVF3^R^. The micrograph (A) shows the typically observed cell morphotypes of *S*. *marcescens* strain LVF3^R^. Micrograph (B) shows *S*. *marcescens* LVF3^R^ surrounded by its active prophages. Cells were grown for 24 h at 30°C in TSB-10 medium, negatively stained and used for TEM analysis.

Grown on TSA-10 medium agar LVF3^R^ revealed round cream-white colonies with an average diameter of 0.340 mm (S3 Fig). A Gram staining of LVF3^R^ resulted in pink stained cells (S4 Fig), indicating a Gram-negative type. The cells’ size ranged from 1–1.6 μm, with epileptic and short cells or straight rods with rounded ends (Fig 1A). The isolate displays a typical morphological characteristic of the *Serratia* genus, such as motility by means of polar flagella, a cell size that ranges from 0.9–2.0 μm and rod-shaped cells with rounded ends [3]. Further, phage particles, presumably originating from activated prophages, could be observed in the bacterial culture (Fig 1B).

### Physiological characterization

**Fig 2:**
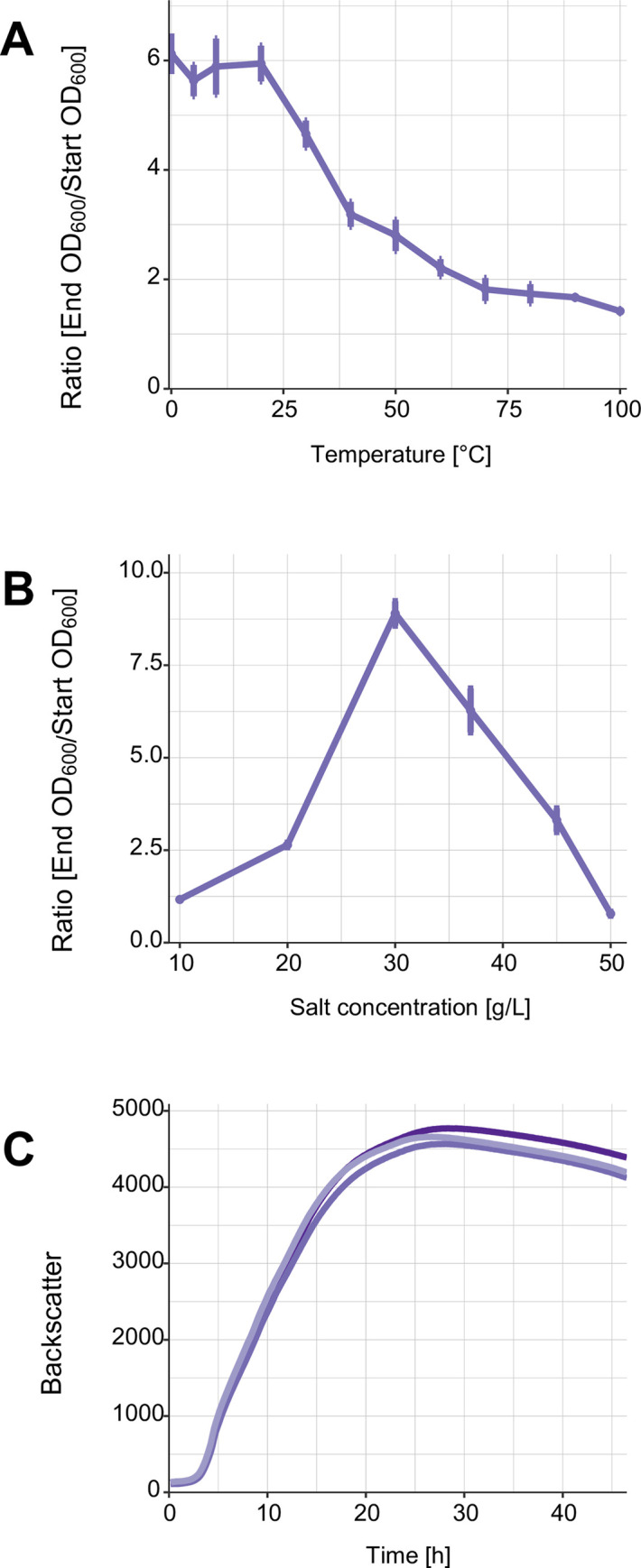
*S*. *marcescens* LVF3^R^ growth properties. (A) Growth of LVF3^R^ in 4 mLTSB medium with different salt concentrations after 3 h incubation at 180 rpm and 30°C. (B) LVF3^R^ growth in TSB-10 medium at different temperatures after 3 h incubation at 180 rpm. (C) Growth analysis of LVF3^R^ at the optimum temperature (30°C) in 25 mL TSB-10 medium. Measurements were performed in triplicate. The standard deviation in (A) and (B) is shown as error bars. In (C) different shades of purple indicate each replicate.

LVF3^R^ showed growth up to 10% (w/v) NaCl in TSB medium with an optimum between 0–2% (w/v) NaCl (Fig 2A). The LVF3^R^ strain was able to grow at a temperature range between 20 and 40°C, which is indicative of a mesophilic organism. The highest cell densities were observed at 30°C with an OD_600_ of 2.670 (which is a ratio of 8.9) (Fig 2B). This observation is in good agreement with data obtained from related strains [3].

The growth kinetics of LVF3^R^ were determined under optimal salt and temperature conditions (Fig 2C). The isolate enters the log phase after a lag phase of approximately three hours which continued for 12 hours until entering a transient phase with reduced growth. Maximum cell densities were observed after around 28 h of cultivation. After the culture reached its peak of maximum growth, cell densities decline, indicating cell-lysis. LVF3^R^ has a doubling time of 304 minutes and a growth rate μ of 0.14 h^-1^.

The metabolic capabilities of LVF3^R^ were analyzed by using the API ZYM and the API 20 E tests. Twenty different enzyme activities were determined via API ZYM for the *S*. *marcescens* isolate. In 13 cases, no enzymatic activity could be determined. The remaining enzymes activities comprised alkaline phosphatase, esterase, esterase lipase, leucine arylamidase, acid phosphatase, naphthol-AS-BI-phosphohydrolase and β-galactosidase activity. Enzymes such as alkaline phosphatase and β-galactosidase were confirmed in the genome playing a role in signaling and cellular processes as well galactose metabolism were confirmed by genome analysis (S1 Table). The alkaline phosphatase is part of the periplasm, whereas the β-galactosidase is part of the outer membrane in *Serratia marcescens* [53]. Interestingly, strain LVF3^R^ is able to utilize urea, which has previously only been described in *Serratia ureilytica* [54]. LVF3^R^ was oxidase-negative and catalase-positive, which is characteristic for the *Yersiniaceae* family [1]. A general overview of all enzymatic activities of the strain and closely related strains from TYGS [36] is listed in Table 1. LVF3^R^ is capable of D-glucose fermentation/oxidation. The antibiogram (S5 Fig) showed that LVF3^R^ is resistant to ampicillin (25 μg/disc), erythromycin (10 μg/disc), oxytetracycline (30 μg/disc), rifampicin (2 μg/disc), tetracycline (30 μg/disc), vancomycin (30 μg/disc), oxacillin (256 μg/disc) and meropenem (until 0.06 μg/disc). Resfams in silico analysis [42] identified genes encoding an ABC transporter for erythromycin or vancomycin (PRJNAA669584|IM817_08890), an MFS transporter for tetracycline or oxytetracycline (IM817_13485), β-lactamases for meropenem (IM817_13270), oxacillin and ampicillin inactivation (IM817_09360), and an efflux pump system of the RND family putatively exporting rifampicin (IM817_09370; S2 Table). The antibiogram as well the congruent in silico investigation of strain LVF3^R^, showed a resistance potential to medically relevant antibiotics. *Serratia marcescens* LVF3^R^ shows a different antibiogram compared to its phylogenetically closest relatives. It is not resistant to chloramphenicol, doxycycline, kanamycin, meropenem and streptomycin. Like *S*. *marcescens* DSM 17174^R^ and *S*. *nematodiphila* DSM 21420^T^, LVF3^R^ is not resistant to chloramphenicol, kanamycin, and streptomycin. Ampicillin and oxacillin resistance seem to be unique to our isolate (Table 1).

**Table 1:** Phenotypic characteristics of strain LVF3^R^ and phylogenetically related species *Serratia* sp. S119^R^, *S marcescens* ATCC 13880^T^, *S*. *marcescens* DSM 17174^R^, *S*. *nematodiphila* DSM 21420^T^.

Characteristics	*S*.* marcescens* LVF3^R^	*Serratia* sp. S119^R^	*S*.* marcescens* ATCC 13880^T^	*S*.* marcescens* subsp. *sakuensis* KCTC 42172^T^	*S*.* nematodiphila* DSM 21420^T^
**Source of isolation**	**Surface water**	Peanut nodule	Pond water	Activated sludge	Intestine of nematode
**Spore formation**	–	–	–	+	–
**Red colony pigmentation**	–	–	+	+	+
**Motility**	+	+	+	+	+
**Glucose oxidation**	+	n/a	n/a	n/a	n/a
**Glucose fermentation**	+	n/a	n/a	+	n/a
**Temperature (°C)**					
Range	10–45	n/a	n/a	n/a	4–42
Optimum	30	28	30–37	28–37	33.5
**NaCl (g/L)**					
Range	0–60	n/a	n/a	0–70	20–70
Optimum	0–20	10	5	5	45
**Utilization of**					
2-nitrophenyl-βD-galactopyranoside	+	+	+	n/a	n/a
L-arginine	–	–	–	+	+
L-lysine	+	+	+	+	+
L-ornithine	+	+	+	+	+
Trisodium citrate	+	+	+	+	+
Sodium thiosulfate	–	–	–	–	–
Urea	+	–	–	–	–
L-tryptophane	–	–	–	+	+
L-tryptophane (indole production)	–	–	v	–	–
Sodium pyruvate (Voges Proskauer)	+	–	v	+	+
Gelatin	+	+	+	n/a	+
D-glucose	+	+	+	+	+
D-mannitol	+	+	+	n/a	+
Inositol	+	+	v	n/a	n/a
D-sorbitol	+	+	+	+	+
L-rhamnose	+	–	v	n/a	n/a
D-sucrose	+	+	+	+	+
D-melibiose	+	+	+	–	+
Amygdalin	+	+	+	n/a	n/a
L-arabinose	–	+	v	–	+
**Catalase**	+	n/a	n/a	+	+
**Oxidase**	–	–	–	–	–
**Resistance to**					
Ampicillin	+	n/a	–	–	–
Chloramphenicol	–	+	+	–	–
Doxycycline	–	n/a	+	n/a	n/a
Erythromycin	+	n/a	+	+	n/a
Kanamycin	–	n/a	+	–	–
Meropenem	–	n/a	n/a	n/a	–
Oxacillin	+	n/a	–	n/a	n/a
Oxytetracycline	+	n/a	n/a	n/a	n/a
Rifampicin	+	n/a	n/a	n/a	–
Tetracycline	+	n/a	+	+	–
Streptomycin	–	n/a	n/a	–	–
Vancomycin	+	n/a	–	n/a	+
**G + C %**	59.29	59.85	59.8	58	59.52

In bold: Sorted by categories.

Taxa: 1, strain *S*. *marcescens* LVF3^R^; 2, *Serratia* sp. S119^R^ (data from [55]); 3, S. marcescens ATCC 13880^T^ (data from BacDive [56] on 24 February 2021); 4, *S*. *marcescens* subsp. *sakuensis* KCTC 42172^T^ (data from [6,57]; LPSN [58] accessed on 24 February 2021); 5, *S*. *nematodiphila* DSM 21420^T^ (data from [57]; BacDive [56] accessed on 24 February 2021); +, Positive; -, negative; v, some strains showed activity; n/a, not available.

Interestingly, non-pigmented strains of *S*. *marcescens* are usually more resistant to antibiotics than pigmented strains as they often harbor resistance plasmids [59]. No potential genes encoding antibiotic resistance were detected in the plasmid sequence of strain LVF3^R^. Environmental *Serratia marcescens* strains are resistant to colistin, cephalothin, ampicillin, tetracycline, and nitrofurantoin [3]. Strain LVF3^R^ does not produce the red-pigmented antibiotic prodigiosin. This is in agreement with the genome analysis as genes of the *pig* cluster encoding the biosynthesis of prodigiosin [60]. were not detected. In a study by Haddix & Shanks (2018), pigmented cells were shown to have twice the biomass yield of non-pigmented *S*. *marcescens* strains [61]. Furthermore, LVF3^R^ appears to produce secondary metabolites such as the antibiotic andrimid, the cyclic lipopeptide orfamide and the O-antigen of lipopolysaccharides (S3 Table). Andrimid production was also detected in the plant-associated *Serratia plymuthica* A153 and *Serratia marcescens* MSU97 [62,63]. Orfamide as a bioactive compound may be released for plant protection [64]. The O-antigen of lipopolysaccharides is responsible for a normal growth rate in plants such as tomatoes [65].

Comparisons of the LVF3^R^ genome to the genomes of the phylogenetically most closely related strain *Serratia* sp. S119^R^ and the PGPR strain *Serratia marcescens* UENF-22GI showed that they share numerous plant-growth promoting genes (49 with S119^R^ and 11 with UENF-22GI). These genes code for components of indole acetic acid (IAA) biosynthesis, siderophore production, plant polymer degradation enzymes, acetoin synthesis, flagellar proteins, type IV secretion system, chemotaxis, phosphorous solubilization, and biofilm formation (S4 Table). All of these genes are known to provide important plant growth-promoting properties [55,66]. *Serratia* sp. S119^R^, a known biofertilizer for peanut and maize, is closely related to LVF3^R^ with 96.08% average nucleotide identity (S5 Table). Based on these results, the environmental origin of isolation (surface water near frog’s lettuce) and the detected physiological properties, it is indicated that *Serratia marcescens* strain LVF3 has the potential to promote plant growth.

### Genomic characterization

#### Genome

Genome sequencing using Illumina and Oxford Nanopore technologies resulted in a high-quality closed genome (S6 Table). The genome of LVF3^R^ consists of one circular chromosome (5,440,698 bp) with a GC-content of 59.29% and one circular plasmid (87,710 bp) with a GC-content of 53.27%. The difference in GC content (6.02%) suggests that the plasmid was obtained recently. The chromosome has a 285.9-fold and the plasmid a 418.7-fold coverage, implying that the plasmid is present in two copies per cell. The chromosome encodes 5,159 protein-encoding genes, 129 rRNAs and 92 tRNAs. The plasmid encodes 94 protein-encoding genes. No CRISPR regions were detected. Genomic characteristics are listed in Table 2.

**Table 2:** Genome statistics of the LVF3^R^ chromosome and p87710 plasmid.

Features	Chromosome	Plasmid
Genome size (bp)	5,440,698	87,710
GC content (%)	59.29	53.27
Coverage	285.9-fold	418.7-fold
CDS	5,159	94
rRNA genes	129	0
tRNA genes	92	0
ncRNA	15	0
CRISPR	0	0
Prophage(s)	2	0

#### Whole-genome phylogeny

**Fig 3:**
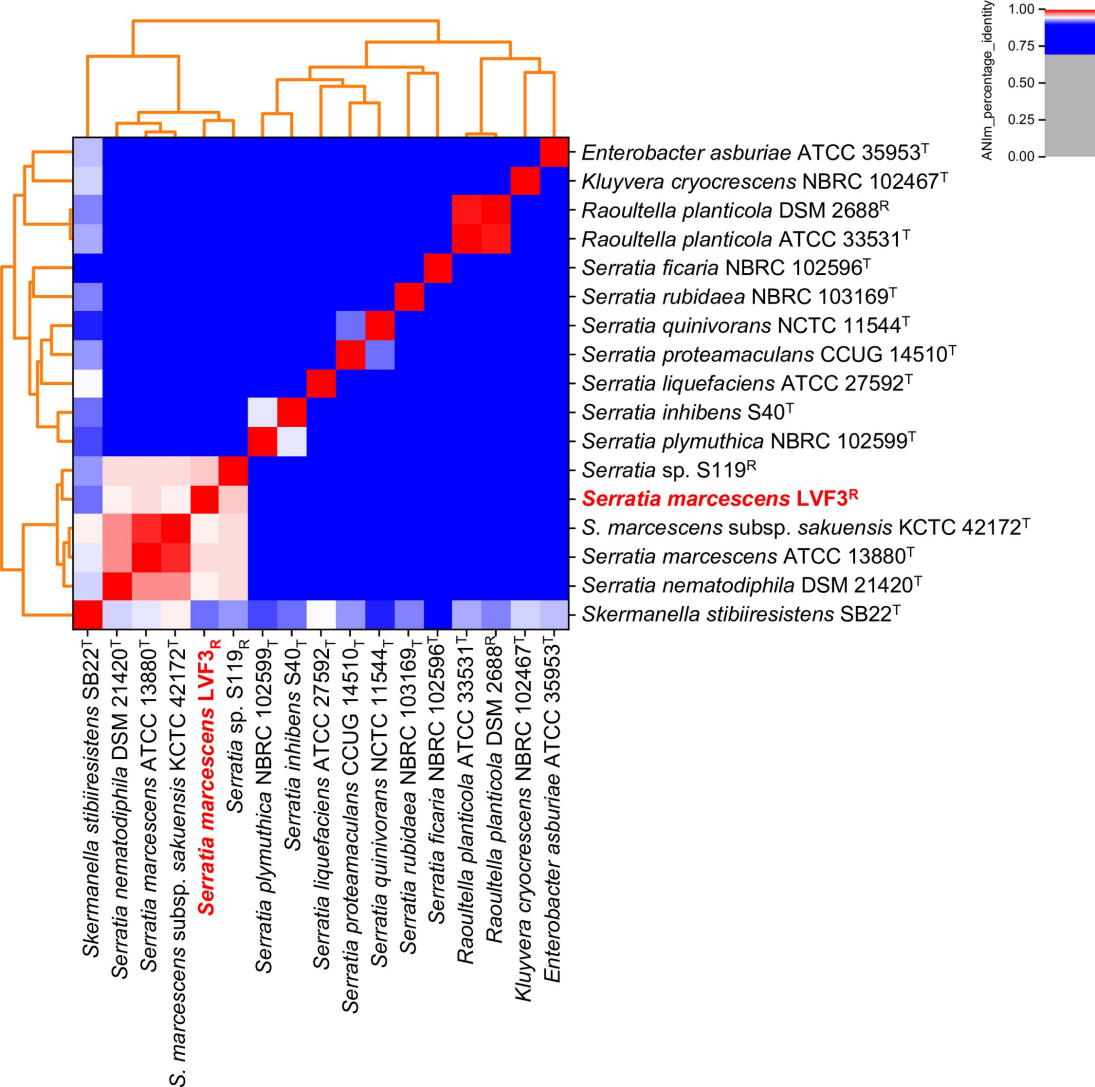
Genome-based phylogenetic analysis of *Serratia marcescens* LVF3^R^. All genomes from available type strains (T) included in the TYGS database [36] and a representative strain (R) from the genus *Serratia* were examined. Calculations were done with pyani [37,68] using ANIm method with default parameters. LVF3^R^ is depicted in bold red.

Initial taxonomic assignment of strain LVF3^R^ was performed with GTDB-Tk pipeline [35]. It revealed an average nucleotide identity (ANI) of approximately 96% to the closest related species *Serratia marcescens* (ANI value of 96.3). This supports LVF3^R^’s assignment to the species *S*. *marcescens* (S6 Table). However, taxonomic assignment of LVF3^R^ employing the Type Strain Genome Server (TYGS) suggests that our strain is a potential new species, although the calculated digital DNA-DNA hybridization (dDDH) value is 73.3%, showing close relationship with the type strain *Serratia marcescens* ATCC 13880 (S1 Fig; S7 Table). The threshold for a new species is below 70% dDDH [67]. ANI-analysis using the 15 closest related type strain genomes derived from the TYGS database [36] as well as the genome of the reference strain (*Serratia* sp. S119) is shown in Fig 3 (data in S6 Table).

The genome of strain LVF3^R^ builds a cluster with the type strains *S*. *marcescens* ATCC 13880, *S*. *nematodiphila* DSM 21420 and *S*. *marcescens* subsp. *sakuensis* KCTC 42172, and the reference strain *Serratia* sp. S119. LVF3^R^ shares the closest average nucleotide identity with *Serratia* sp. S119^R^ (96.08%) and *S*. *marcescens* ATCC 13880^T^ (95.33%).

Based on the results of TYGS, GTDB-Tk and ANI analyses, we suggest that strain LVF3 belongs to the species *Serratia marcescens*.

#### Prophages

The prophage potential of LVF3^R^ was of particular interest as the strain represents a potential host system for studying phage diversity in the environment. Prophage region were initially analyzed using PHASTER [41], revealing two putative prophage regions (region 1: 2,088,804–2,147,829; region 2: 2,353,448–2,400,701). The regions comprised 59.0 and 47.2 kb and were classified as intact (S7 Table).

**Fig 4:**
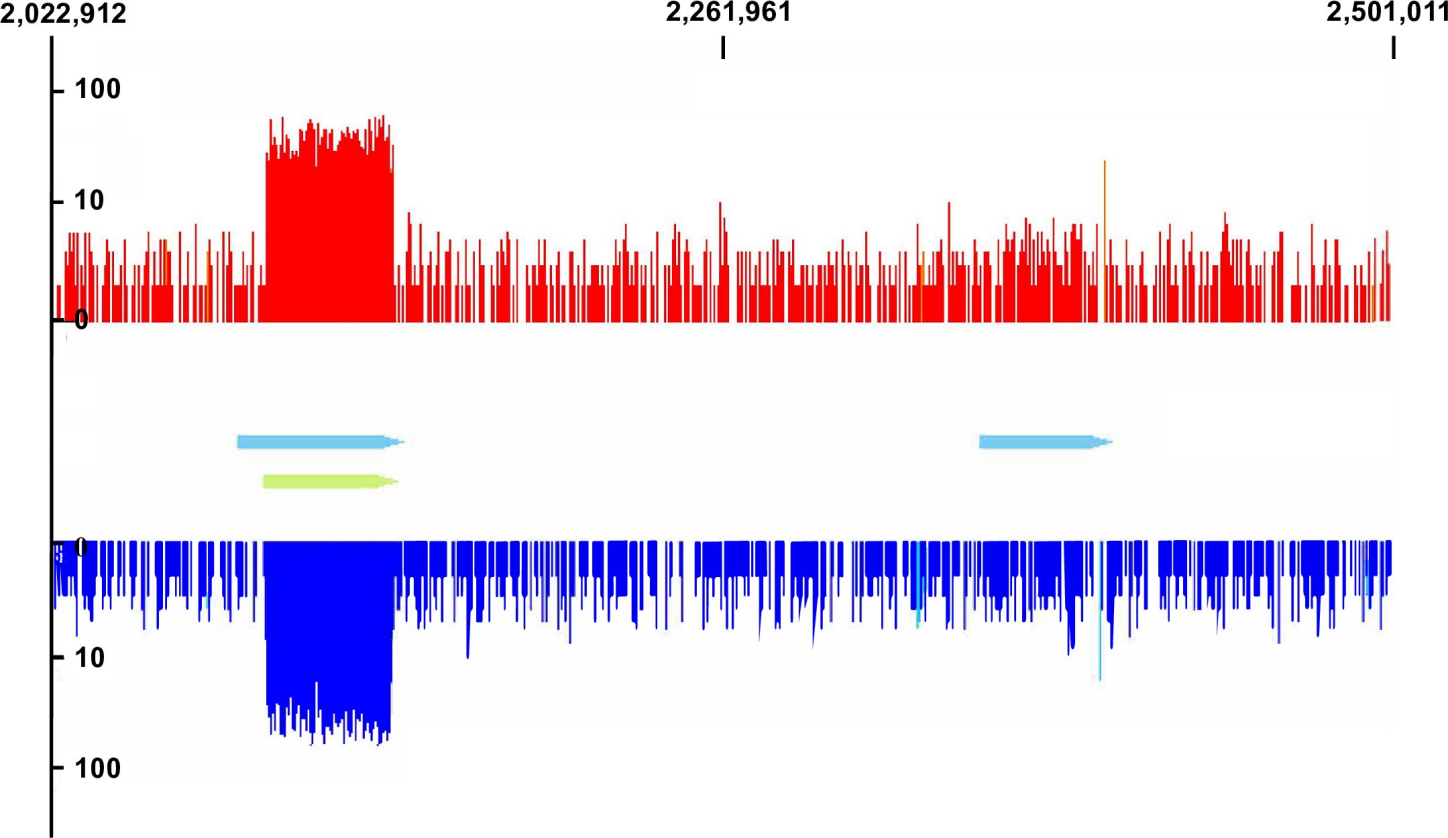
Read coverage profile of sequenced LVF3^R^ prophages, mapped onto its corresponding host genome. The blue arrows depict the prophage regions predicted by PHASTER [41]. Green arrows indicate the experimentally verified prophage region. The image displays the read coverage of the genome between base 2,022,912 to 2,5011,011 (478,099 kb).

Sequence data of phage particle-packed dsDNA was mapped to the LVF3^R^ genome using ProphageSeq [69] (Fig 4). Prophage activity is indicated when prophage reads accumulate closely associated with the PHASTER-predicted prophage regions. The coverage profile exhibits an even distribution of reads with a substantial coverage increase from base 2,089,081 to 2,143,727 (Fig 4). As the PHASTER-predicted prophage region one was annotated with a preceding start site, the precise location of prophage one was investigated. Reads obtained from particle-packed dsDNA were used for genome assembly. This resulted in one circular contig with a size of 45,631 bp representing the phage genome of the identified prophage. Comparison of the phage genome with the chromosome of LVF3^R^ enabled us to precisely locate the corresponding prophage region. Thus, prophage one is located between 2,098,352 and 2,144,007 bp flanked by perfect direct repeats of 25 bp (5’ AGGAATCGTATTCGGTCTTTTTTTG), which represented the *attL* and *attR* sites. For prophage two, neither a pronounced sequence accumulation was observed at the predicted prophage region, nor was it possible to assemble the respective phage genome.

In conclusion, one prophage region was experimentally confirmed as particle-forming and capable of packing its genome. The second predicted prophage was unable to form particles under the employed experimental conditions. As prophages can mediate resistance against related phages, a low number or absence of prophages in the genome is required for a potential host strain employed for phage isolation [23] and covering viral diversity in an isolation experiment.

#### Strain suitability for phage isolation

**Fig 5:**
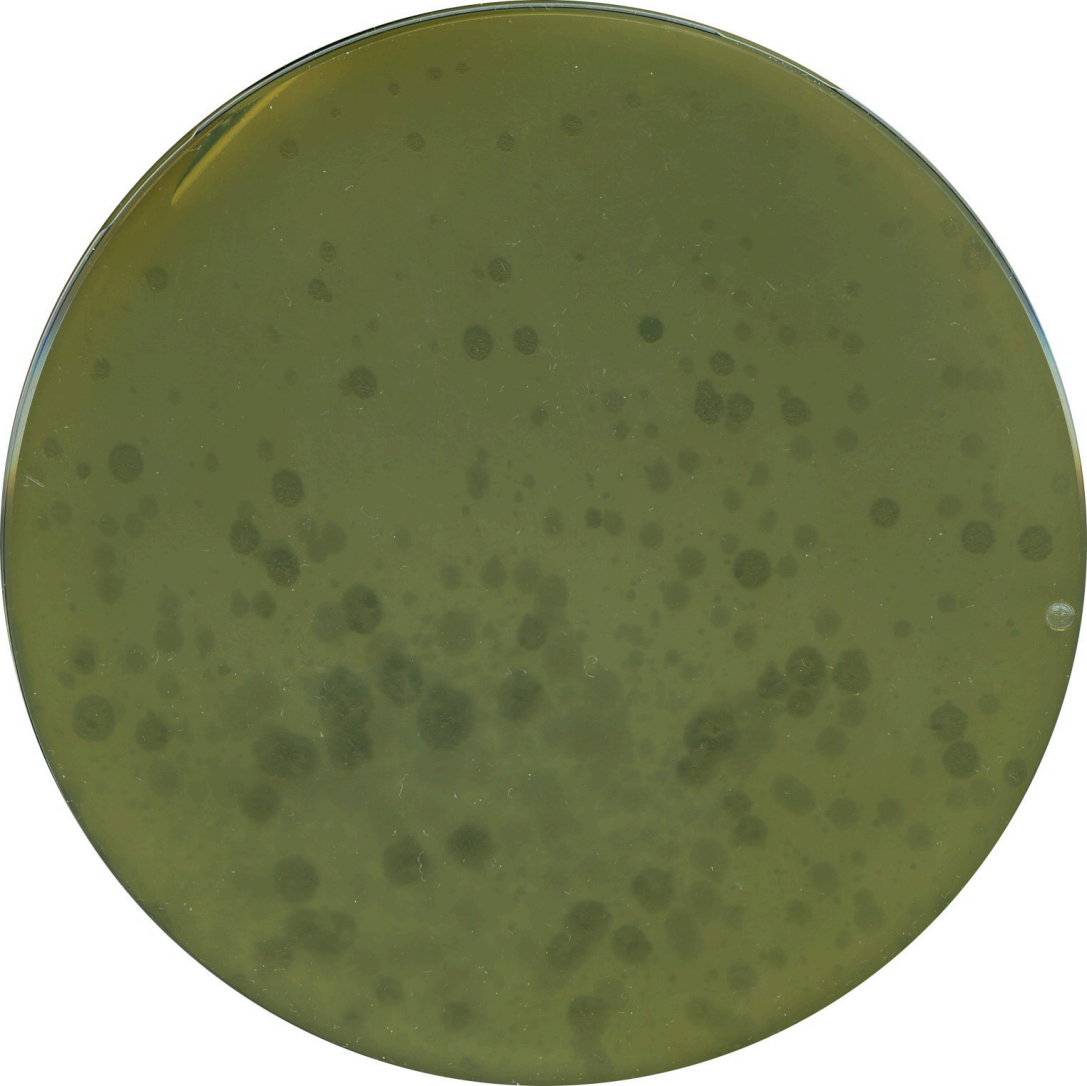
Host strain LVF3 challenged with metaviral sample. Different plaque morphologies can be observed.

So far, *S*. *marcescens* strain LVF3^R^ has proven to be an easy-to-cultivate organism with simple growth requirements and few intrinsic antibiotic resistances. This provides a good basis for making it a potential working strain in molecular biology. In a next step, we aimed to assess its potential as host strain for environmental phage isolations. For this purpose, LVF3^R^ was infected with a viral suspension derived from raw sewage. An overlay plaque assay was employed to analyze the infected cells (Fig 5). Results revealed diverse plaque morphologies corresponding to different phages, thus confirming the suitability of *S*. *marcescens* LVF3^R^ as a host strain for phage isolation,

## Conclusion

In the framework of this study, the novel *Serratia* strain LVF3R was characterized and determined to be a suitable host strain for environmental phage isolation as it only contains one active and one degenerated prophage in its genome. Further, we could confirm that our strain showed after infection with a viral pool, a high phage diversity. The viral diversity associated with this strain will be the subject of future studies.

## Supporting information

S1 Fig Phylogenetic classification of *Serratia marcescens* strain LVF3. (PDF)

S2 Fig Visualization of functional categories through BlastKoala (Kanehisa et al., 2016) [38] for *Serratia marcescens* LVF3^R^. Functional categories of (A) chromosome and (B) plasmid can be seen by the presented color code.
(PDF)

S3 Fig Colony morphotype of *Serratia marcescens* LVF3^R^. Growth experiments were performed using TSA-10 agar plates.
(PDF)

S4 Fig Gram staining of *Serratia marcescens* LVF3^R^. (PDF)

S5 Fig Analysis of antibiotic resistances through soft-agar assay with discs (A) and strips (B). Exemplarily, antibiotic resistance of isolate LVF3R is indicated by halo formation. Incubation took place overnight at 30°C. (A) Meropenem (0.002–32 μg), and (B) kanamycin (30 μg), chloramphenicol (30 μg), streptomycin (10 μg) and rifampicin (2 μg) were used as antibiotics.
(PDF)

S1 Table KEGG Mapper Reconstruction Result of *Serratia marcescens* LVF3^R^. (XLSX)

S2 Table Resfams prediction of *Serratia marcescens* LVF3^R^. (XLSX)

S3 Table List of putative biosynthetic gene clusters in *Serratia marcescens* LVF3^R^. (XLSX)

S4 Table Comparison of putative genes involved in important plant growth promoting traits of *Serratia marcescens* LVF3R, *Serratia* sp. S119^R^ and *Serratia marcescens* UENF-22GI. Table was modified from Ludueña et al., 2017 and Matteoli et al., 2018. In purple: Potential plant growth promoting gene products encoded by the genome of LVF3^R^. References: Ludueña LM, Anzuay MS, Angelini JG, McIntosh M, Becker A, Rupp O, et al. Strain Serratia sp. S119: A potential biofertilizer for peanut and maize and a model bacterium to study phosphate solubilization mechanisms. Appl Soil Ecol. 2017;126:107–12. Matteoli FP, Passarelli-Araujo H, Reis RJA, da Rocha LO, de Souza EM, Aravind L, et al. Genome sequencing and assessment of plant growth-promoting properties of a Serratia marcescens strain isolated from vermicompost. BMC Genomics. 2018;19:750.
(XLSX)

S5 Table Phylogenetic analysis for *Serratia marcescens* LVF3^R^. (XLSX)

S6 Table GTDB-Tk of *Serratia marcescens* LVF3^R^ isolate. (XLSX)

S7 Table Pairwise comparisons of LVF3 against type strain genomes from TYGS (Meier-Kolthoff and Göker, 2019). Reference: Meier-Kolthoff JP, Göker M. TYGS is an automated high-throughput platform for state-of-the-art genome-based taxonomy. Nat Commun. 2019;10:2182.
(XLSX)

S8 Table PHASTER analysis of *Serratia marcescens* LVF3^R^. (XLSX)
