# Supplementary material for: Down in the pond: Isolation and characterization of a new Serratia marcescens strain (LVF3) from the surface water near frog’s lettuce (Groenlandia densa)
Source: PLoS One. 2021 Nov 8;16(11):e0259673. doi: 10.1371/journal.pone.0259673 (PMC8575298; doi:10.1371/journal.pone.0259673)
Supplement: S2 Fig — Functional categories of (A) chromosome and (B) plasmid can be seen by the presented color code. (PDF) [file pone.0259673.s002.pdf]

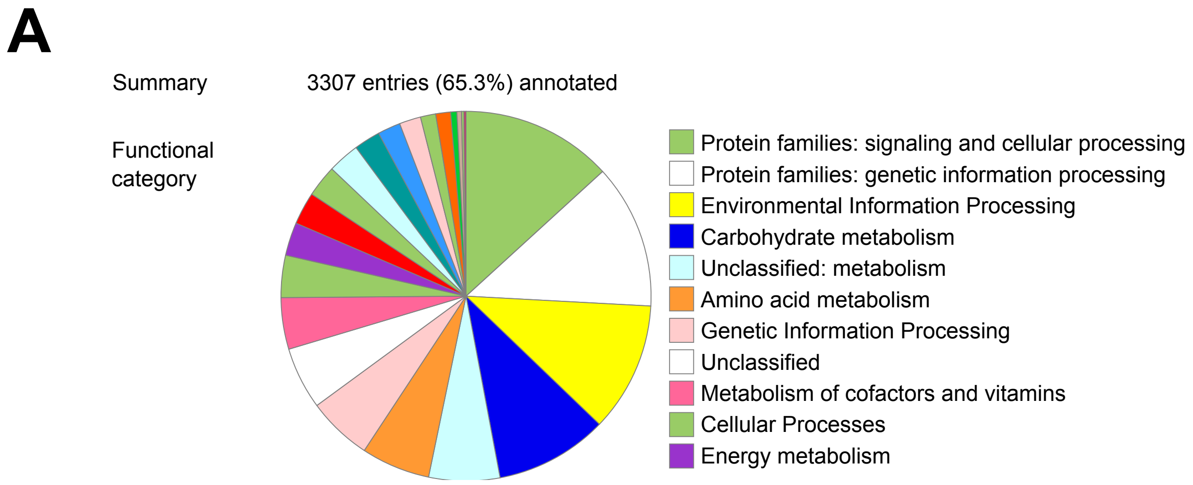

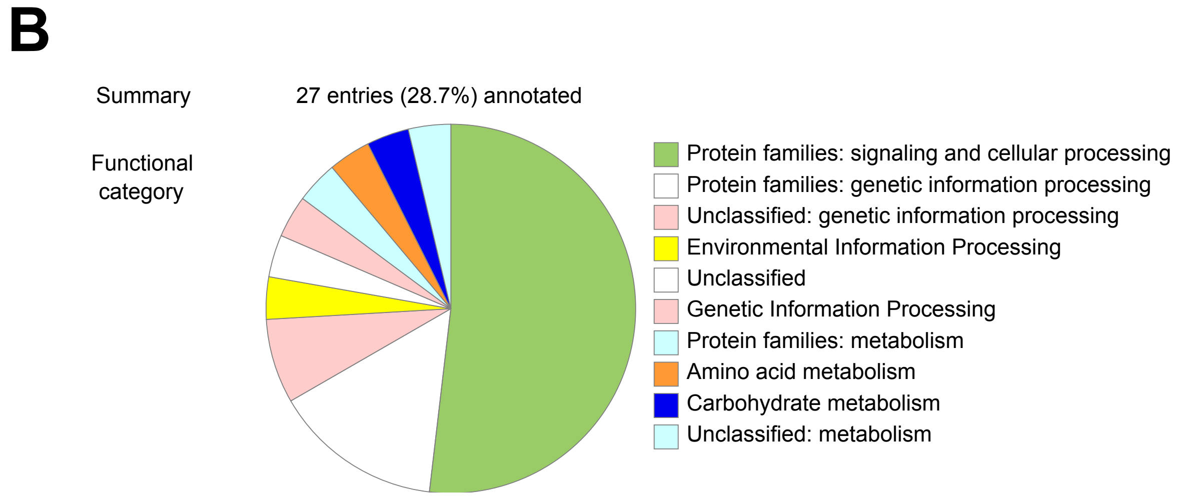

S2 Fig. Visualization of functional categories through BlastKoala (Kanehisa et al., 2016) **for *Serratia marcescens* LVF3^{R}.** Functional categories of (A) chromosome and (B) plasmid can be seen by the presented color code .

Reference:

Kanehisa M, Sato Y, Morishima K. BlastKOALA and GhostKOALA: KEGG tools for functional characterization of genome and metagenome sequences. J Mol Biol. 2016;428:726–31.
