# Supplementary material for: Down in the pond: Isolation and characterization of a new Serratia marcescens strain (LVF3) from the surface water near frog’s lettuce (Groenlandia densa)
Source: PLoS One. 2021 Nov 8;16(11):e0259673. doi: 10.1371/journal.pone.0259673 (PMC8575298; doi:10.1371/journal.pone.0259673)
Supplement: S3 Fig — Growth experiments were performed using TSA-10 agar plates. (PDF) [file pone.0259673.s003.pdf]

Isolate	Solid culture
Serratia marcescens **LVF3^{R}**	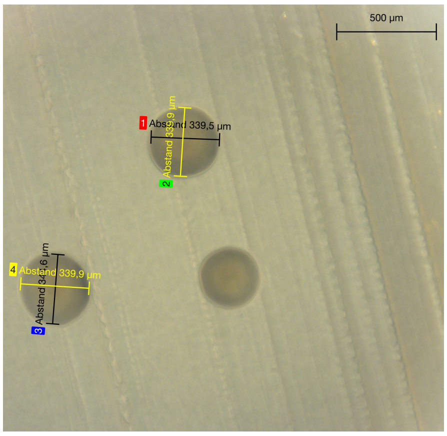

S3 Fig. Colony morphotype of *Serratia marcescens* LVF3^{R}. Growth experiments were performed using TSA-10 agar plates.
