# Supplementary material for: Down in the pond: Isolation and characterization of a new Serratia marcescens strain (LVF3) from the surface water near frog’s lettuce (Groenlandia densa)
Source: PLoS One. 2021 Nov 8;16(11):e0259673. doi: 10.1371/journal.pone.0259673 (PMC8575298; doi:10.1371/journal.pone.0259673)
Supplement: S5 Fig — Analysis of antibiotic resistances through soft-agar assay with discs (A) and strips (B). Exemplarily, antibiotic resistance of isolate LVF3R is indicated by halo formation. Incubation took place overnight at 30°C. (A) Meropenem (0.002–32 μg), and (B) kanamycin (30 μg), chloramphenicol (30 μg), streptomycin (10 μg) and rifampicin (2 μg) were used as antibiotics. (PDF) [file pone.0259673.s005.pdf]

A

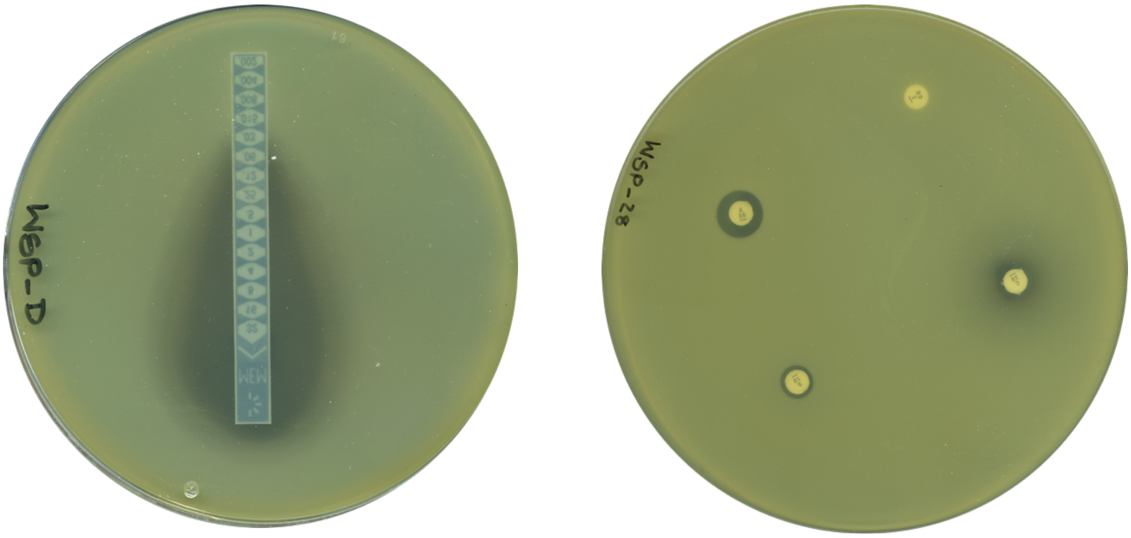

S5 Fig. Analysis of antibiotic resistances through soft-agar assay with discs (A) and strips (B). Exemplarily, antibiotic resistance of isolate LVF3^{R} is indicated by halo formation. Incubation took place overnight at 30 °C. (A) Meropenem (0.002–32 µg), and (B) kanamycin (30 µg), chloramphenicol (30 µg), streptomycin (10 µg) and rifampicin (2 µg) were used as antibiotics.
